# Fractional Dynamics of Network Growth Constrained by Aging Node Interactions

**DOI:** 10.1371/journal.pone.0154983

**Published:** 2016-05-12

**Authors:** Hadiseh Safdari, Milad Zare Kamali, Amirhossein Shirazi, Moein Khalighi, Gholamreza Jafari, Marcel Ausloos

**Affiliations:** 1 Department of Physics, Shahid Beheshti University, G.C., Evin, Tehran 19839, Iran; 2 Department of Mathematics, Tarbiat Modares University, Tehran, Iran; 3 The Institute for Brain and Cognitive Science (IBCS), Shahid Beheshti University, G.C., Evin, Tehran 19839, Iran; 4 Center for Network Science, Central European University, Nador 9, 1051 Budapest, Hungary; 5 GRAPES, rue de la Belle Jardiniere 483, B-4031, Angleur, Belgium; 6 School of Management, University of Leicester, University Road, Leicester, LE1 7RH, United Kingdom; 7 eHumanities group, Royal Netherlands Academy of Arts and Sciences, Joan Muyskenweg 25, 1096 CJ, Amsterdam, The Netherlands; IFIMAR, UNMdP-CONICET, ARGENTINA

## Abstract

In many social complex systems, in which agents are linked by non-linear interactions, the history of events strongly influences the whole network dynamics. However, a class of “commonly accepted beliefs” seems rarely studied. In this paper, we examine how the growth process of a (social) network is influenced by past circumstances. In order to tackle this cause, we simply modify the well known preferential attachment mechanism by imposing a time dependent kernel function in the network evolution equation. This approach leads to a fractional order Barabási-Albert (BA) differential equation, generalizing the BA model. Our results show that, with passing time, an aging process is observed for the network dynamics. The aging process leads to a decay for the node degree values, thereby creating an opposing process to the preferential attachment mechanism. On one hand, based on the preferential attachment mechanism, nodes with a high degree are more likely to absorb links; but, on the other hand, a node’s age has a reduced chance for new connections. This competitive scenario allows an increased chance for younger members to become a hub. Simulations of such a network growth with aging constraint confirm the results found from solving the fractional BA equation. We also report, as an exemplary application, an investigation of the collaboration network between Hollywood movie actors. It is undubiously shown that a decay in the dynamics of their collaboration rate is found, even including a sex difference. Such findings suggest a widely universal application of the so generalized BA model.

## Introduction

The history of events plays a crucial role in the dynamics of many political, economic, social, technological, legal and environmental (PESTLE) systems,—in particular due to psychological phenomena, but also biological and physical conditions. The future choices a system makes are heavily influenced by its past [1]. Although, there are many different models to describe a network evolution, effects of history and age are often not considered in such models. Here, we study the role of the aging process on network growth dynamics, in the context of the Barabási- Albert (BA) model as one of the models of choice for describing network dynamics based on preferential attachment [2–5].

The BA model, inducing hubs in networks, i.e. nodes with a very large number of incoming or/and outgoing links, characterizes one of the important features of real networks, namely, scale invariance [6–10]. Despite being very successful in describing many properties of real networks [2], this model also implies that nodes can have an unlimited number of links,—a feature which is not always in line with reality [11, 12]. In fact, in real networks, other factors work against such a growth of node degrees, which the BA model fails to account for [13–15]. For instance, in realistic networks, like citation and scientific collaboration networks [16, 17], the world-wide web network [18] and network of movie actor collaborations [19], there are important phenomena such as aging [20–24], and related aspects like those found in screening for diffusion limited aggregation [25], limited capacity, as in city size or population growth [26–31], or censorship [32–35], in the spread of knowledge, of friendship on electronic social networks, or in geo-political realms, which are not considered inside the BA model.

In the network of movie actors, overtime “superstars” (equivalent to “movie network hubs”) are replaced by new ones. Indeed, superstars get promoted for some period of time but eventually lose their attraction (in the eyes of the media), and then their appearance gradually decays. New generations of stars replace the old generations; a similar cycle repeats again. In political networks [36, 37], the influence of people grows and eventually decreases: those who were political class rulers and party life controllers are replaced by often younger people. No one experiences unbound growth in politics. In academia, the number of co-authors of most scientists grows, but later on decays [9]. There is no need to recall further that in the economy, an unlimited company growth, after reaching some large share of the market, saturates and decays (if there is e.g. no innovation or merging) [38]. The argument could surely go on for the 4th type of power network, the military network, as discussed in the IEMP model [39].

The number of active friends a person can have is also limited, because a person can devote only part of his/her time to each of those friends. Since there is a limited amount of time per day/week, one cannot realistically have an infinite number of friends,—whatever a friend means e.g. on Facebook or on Myspace. Those “most important” friends loose to be truly “old friends” because the links age, and because new “potential friends” and subsequently strong links appear [40]. This is similar to the screening effect phenomenon in Fermi systems, in which the electronic potential is reduced by the cloud of electrons around one electron [41], to the capacity limiting effect of Verhulst on population growth [42] or the size dependent competition-cooperation effect in [43, 44].

Thus, in many real systems, each node has a limited “capacity” for joining with other nodes. However, according to the BA model, nodes can connect to each other without any restriction, whence their degree can grow boundlessly. In the BA model a “hub node” will always remain dominant as compared to newcomers, and its role as a hub will increase with time. To adapt the preferential attachment mechanism to some reality, the BA-based model ideas should pay attention to these kinds of effects. A number of studies have been done in this area: for example Palla et. [45] have found that large groups persist longer if they are capable of dynamically altering their membership, suggesting that an ability to change the group composition results in better adaptability. Notice that the behavior of small groups displays the opposite tendency: “the condition for stability is that their composition remains unchanged” [45]. We would go on, suggesting not only that clusters on the network change the number of nodes and their quality, but also attitudes, types of links, should be considered (to be) changing.

Strategies have been proposed based on adding new terms to the BA model [13, 46–48]. It is worth noticing that other recent studies about growing network models include the aging of nodes as a key feature [49, 50]. However, requesting special *ad hoc* behavior is not a suitable approach to introduce history effects; often, the resulting features do not have a closed form solution either.

In order to mimic the age of a node, toward describing some impact on the network growth dynamics, we impose a time dependent kernel on the evolution equation of the node’s degree, within the BA preferential attachment mechanism. A specific power law temporal kernel function has been chosen in order to preserve the effect of old events in comparison to recent ones. Hence, we introduce past information through a weighting function. An event in the far past is supposed to have less effect on the node’s dynamics, when compared with the same event happening in a near past. As a result of this kernel, the governing equation for the node’s dynamics is turned into a fractional [51–56] order BA-like equation.

**Fig 1:**
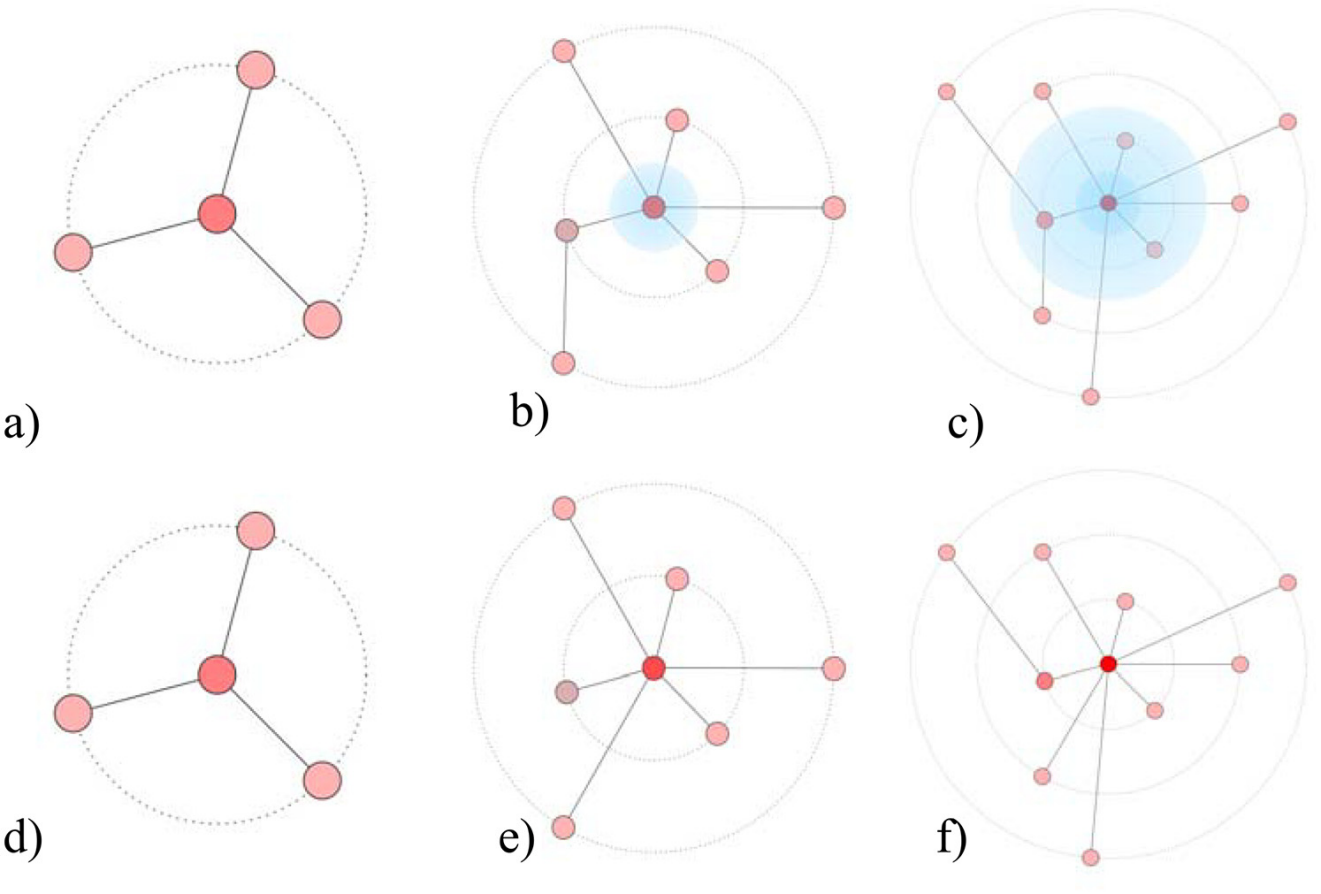
Schematic diagram representing the dynamical growth of nodes in a network, developing under a preferential attachment mechanism, but also considering limitations imposed by an aging process and screening effects on the growth process (a-c); comparisons with standard preferential attachment are found in (e-f). Red to blue color shades of nodes represent the attractiveness for new links. The highest attractiveness corresponds to the red color. The radial distance from the central node indicates the arrival time for a node. Old members, because of their age, have a lower chance of being selected. In (e-f), i.e. in the absence of these effects, hubs dominate the attachment process of new links.

Fig 1 schematically represents the growth process of a network for which its aging process has been considered in the dynamics. It is clear from this illustration that the limiting capacity for receiving new links and the aging of links over time decrease the chance of high degree nodes to be attracting new links.

We have also simulated a network on which old links have less impact on a node’s dynamics when compared with newer links. In fact, a node’s effective degree is calculated by considering the age of each link: thereafter, each node attracts new connections according to its effective degree.

Besides the numerical solution of the differential equation and analyzing the characteristics of the simulated networks, as an actual example, we have studied the network of collaboration between Hollywood movie actors. By following their cooperation dynamics along their career time, the aging effect on this network evolution can be observed. It is found that the actor network dynamics is in close agreement with the presented model, thereby suggesting a wide application range of our model toward many other real cases.

## Methods

### Imposing temporal kernel on preferential attachment algorithm

To take into the account the aging effects, we start with the preferential attachment rule, according to which at each time step a new node joins the system and links to *m* already presented nodes. Each present node can receive a new link with a probability proportional to its degree. Beside this mechanism, we add an aging process, which reduces the effect of each link. Indeed the past degrees of a node are used as weight factors, which account for aging of its links [57–60]. Combination of these two processes, namely, preferential attachment and aging of links, results in a BA like integro-differential equation with a time kernel, *κ*(*t* − *t*′), when integrating over the degree evolution, *dk*_*i*_(*t*). Thus, one can write,

$$\begin{array}{c} \frac{dk_{i}\left(t\right)}{dt}=\int_{t_{0}}^{t}dt^{\prime }\kappa \left(t-t^{\prime }\right)\frac{mk_{i}\left(t^{\prime }\right)}{\sum_{j}^{t^{\prime }}k_{j}\left(t^{\prime }\right)}. \end{array}$$ (1)

The degrees of nodes couple to each other through summation in the denominator over degree of all nodes up to previous time. In case of non-aged systems, the kernel should be a Dirac delta function, *δ*(*t* − *t*′), which would result in an integer order integro-differential equation, as in the BA model for network growth.

Here, we choose a power law functionality for the kernel, for describing aging process. By this choice, over time, far past links lose their effectiveness more compared to nearly connected links. This kernel, guarantees the existence of scaling features as they contain the intrinsic nature of most phenomena [61–63]. Most of the time, a distant past event should have far less effects on present ones, when compared with near past events; the only exceptions are large influential events which even overshadow recent ordinary events. To model these exogenous causes, extra parameters should be introduced [64, 65]. By substituting the power law kernel in Eq (1), one has

$$\begin{array}{c} \frac{dk_{i}\left(t\right)}{dt}=\frac{1}{\Gamma (\alpha -1)}\int_{t_{0}}^{t}dt^{\prime }{\left(t-t^{\prime }\right)}^{\alpha -2}\frac{mk_{i}\left(t^{\prime }\right)}{\sum_{j}^{t^{\prime }}k_{j}\left(t^{\prime }\right)}. \end{array}$$ (2)

From fractional calculus methods, it is apparent that the right hand side of this equation is a fractional integral of order (*α* − 1): ${}_{t_{0}}D_{t}^{-(\alpha -1)}$, on the interval [*t*_0_, *t*] [51]. Therefore, it can be obtained that

$$\begin{array}{c} \frac{dk_{i}\left(t\right)}{dt}{=}_{t_{0}}D_{t}^{-(\alpha -1)}[\frac{mk_{i}\left(t\right)}{\sum_{j}^{t}k_{j}\left(t\right)}]. \end{array}$$ (3)

For *α* = 1, the fractional operator turns to unity operator, ${}_{t_{0}}D_{t}^{0}\equiv 1$, hence standard BA model could be recovered.

Now applying a fractional Caputo derivative of order (*α* − 1) [66] on both sides of the above equation, we can write it in the form of a differential equation,

$$\begin{array}{c} {}_{t_{0}}^{c}D_{t}^{\alpha }[k_{i}\left(t\right)]=\frac{mk_{i}\left(t\right)}{\sum_{j}^{t}k_{j}\left(t\right)}, \end{array}$$ (4)

with each node’s initial degree as *k*_*i*_(*t*_*i*0_) = *m*. In this way, the governing equation is a fractional order differential equation, which guarantees the presence of aging. More details on fractional derivatives and relevant methods for numerical solutions can be found in the appendix. The discrete form of the fractional order differential Eq (4) becomes,

$$\begin{array}{c} k_{n}=k_{0}+h^{\alpha }\Sigma_{j=0}^{n-1}b_{n-j-1}\frac{mk_{j}}{\sum_{j}^{t}k_{j}\left(t\right)}. \end{array}$$ (5)

Here, the *b*_*n*_’s are time dependent coefficients, which measure the aging effect. They are further outlined in the appendix.

These *b*_*n*_ factors indicate the contribution of the previous degree of the *i*-th node toward its present value. With passing time (increasing *n*), each *b*_*n*_ becomes smaller. In other words, over the time, the old links of a node lose their effect on the node growth process due to the *b*_*n*_ coefficients limiting form. Therefore, the effective degree of a node, Eq (5), will decrease. Notice that for *α* = 1, each *b*_*n*_ converges toward unity: one gets back to the standard BA model in which old degrees all have the same weight.

## Results

**Fig 2:**
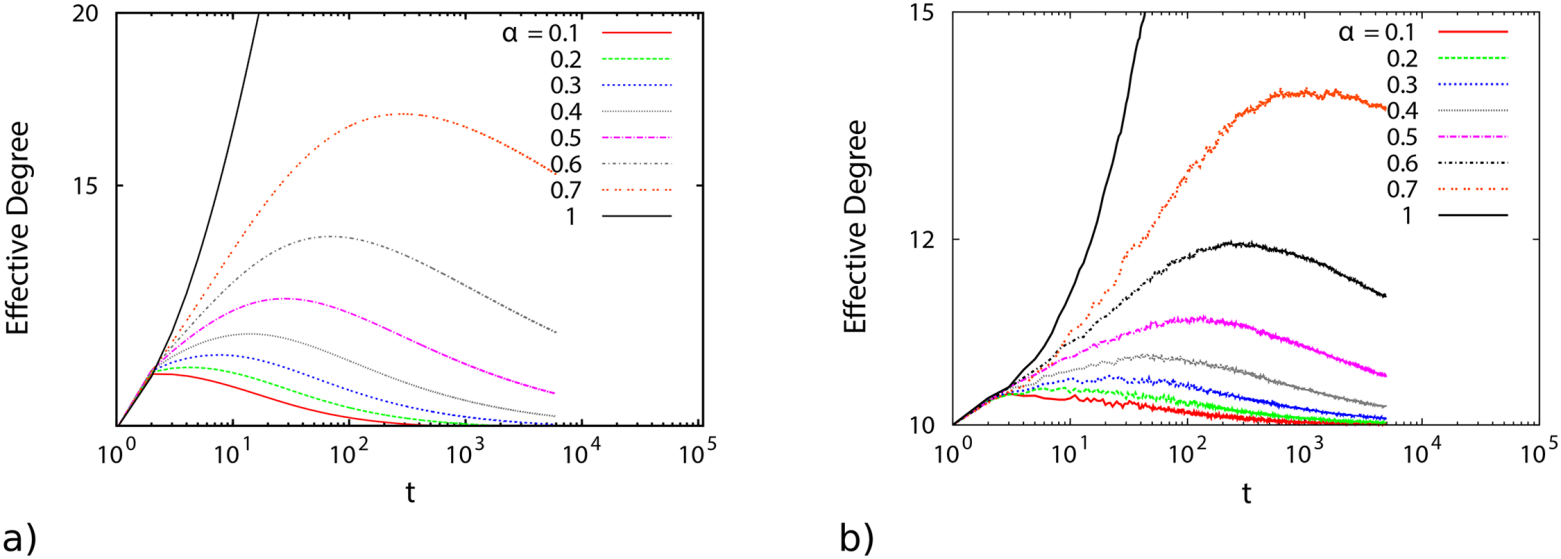
Fig (2a) is the numerical solution of Eq (5) for the mean degree *k*; Fig (2b) is the corresponding mean degree *k* of nodes from network simulation, when its growth dynamics is affected by an aging process. It is clear from such graphs that *k*(*t*) behaves differently from the unlimited growth predicted by the BA model (*α* = 1). Due to the aging process *k*(*t*) reaches a peak and declines gradually afterwards. Such simulations have been performed for 5000 time steps, with as initial condition *m*_0_ = 11 nodes and every new node connecting to *m* = 10 earlier nodes.

**Fig 3:**
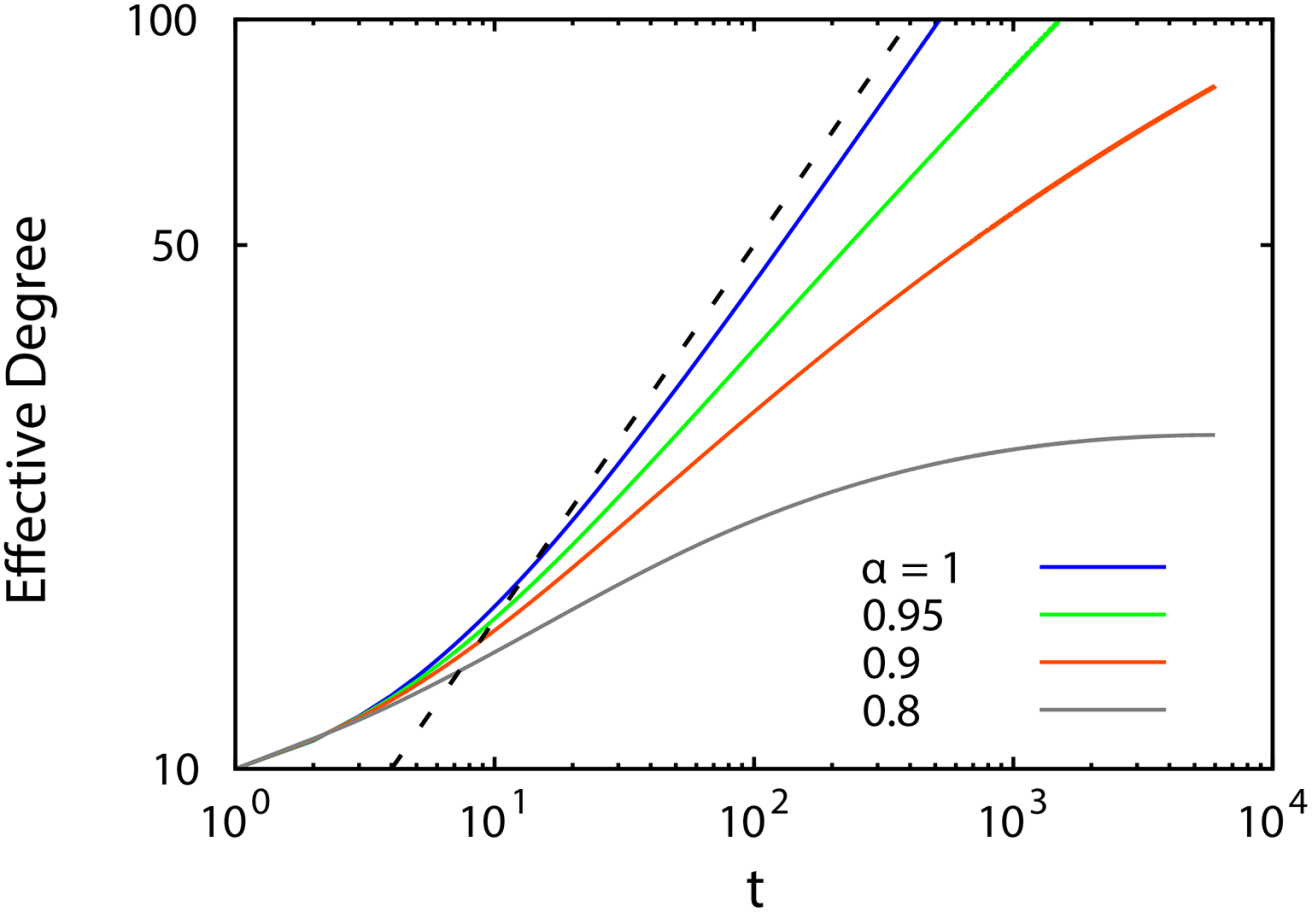
Comparison between the numerical solution of Eq (5) (solid lines) for exponents close to unity and the solution of the standard BA model (dashed line). For long times, the solution of the fractional order equation of network growth dynamics for *α* = 1 tends to the standard BA model.

Numerically solving the equation system, Eq (5), we find the results confirming the effect of aging on the network evolution. Fig 2(a) illustrates the time dependence of the effective degree of a node at time *t* for various *α* values. It is obvious that for all values of *α* < 1, the effective degree of a node *i* increases at first, reaches a maximum value, and then starts to decay. In the preferential attachment mechanism, a node absorbs new links proportional to its degree. However, here, two different processes control the growth dynamics. A node with a high degree has a high chance of receiving new links. However, these nodes usually have more aged links, which makes their effective degree smaller and in turn the probability of new link attachments to such a node decreases. Fig 3 shows the convergence of the asymptotic behavior of the node’s degree in an aged network for *α* = 1 with respect to the corresponding result of the standard BA model.

So far, we have seen the effects of aging on the node’s evolution in a network. In order to see the effects of aging on other usual characteristics of a network, we have simulated an “aged network”. To do this simulation, we start with an initially fully connected network with *m* + 1 nodes. At each time step a node with *m* links is added to the network. We record the link’s age, i.e. the time when a link is added to each of the *m* nodes. New links are connected to already presented nodes, which are selected according to their effective degree. This effective degree is found depending on the age of each link at that time step as,

$$\begin{array}{c} k_{effective}{\left(t\right)}_{i}=\Sigma_{t^{\prime }=1}^{t-1}(b_{\left(t^{\prime }\right)}*dk_{i}\left(t^{\prime }\right)). \end{array}$$ (6)

As should be expected, for the simulated networks, the effective degree experiences a growth followed by some decay, as shown in Fig 2b. This observed behavior is in agreement with Fig 2a, for the numerical solution of the fractional order differential equation, Eq (5).

**Fig 4:**
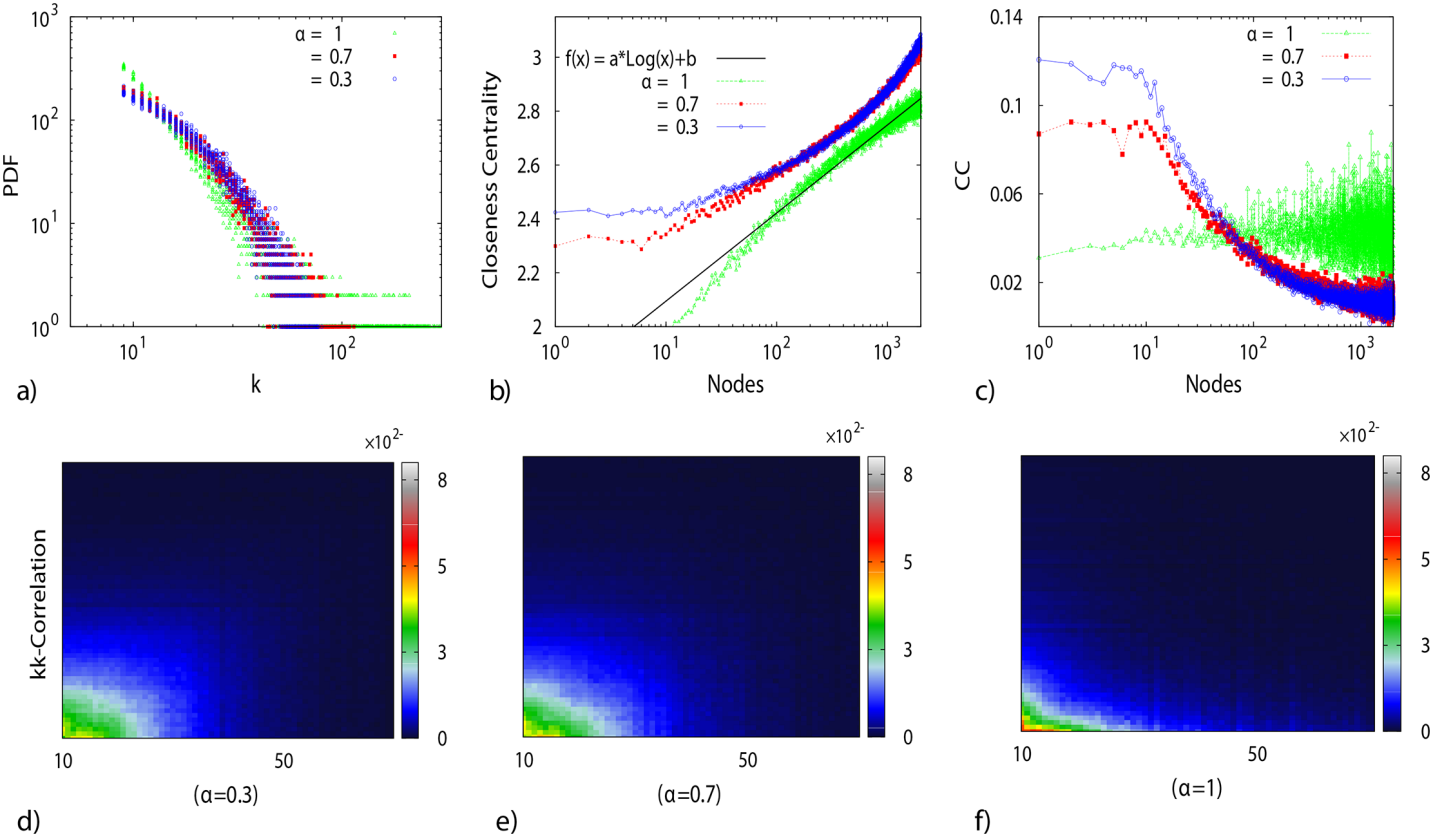
Panel (a) shows the probability distribution function for different orders of the fractional derivative. While for *α* = 1, i.e. the BA model, the expected power law behavior is found, for fractional orders less than unity, there is noticeable deviation from a power law. This could be caused by two competitive processes, namely a preferential attachment mechanism and a screening effect. Panel (b) is the network closeness centrality measure averaged over all nodes; the horizontal axis is the label of nodes according to their arrival time. The comparison for three exponents *α* point to different behaviors. For high values of *α*, this measure has high amounts. Elder nodes have short paths to others. The black solid line shows the fitted curve for *α* = 1. Panel (c) indicates the network clustering coefficient averaged over all nodes for three *α* exponents. The horizontal axis also gives the label of nodes according to their arrival time. Older nodes have more communities around themselves. Panels (d-f) show the degree-degree correlation averaged over all nodes for the three *α* exponents. The tendency to bind with similar members is shown to have been increased by including the effects of past events. The inclination toward linking to previously powerful nodes is reduced. This results from the competition between new generations.

In Fig 4, we also show some statistical proprieties of the simulated networks.

- Recall that the degree distribution is one of the major characteristics of a network. For the preferential attachment model, the degree distribution follows a power law form [2]. We have shown here above that in presence of aging, deviation from a power law occurs, see Fig 4a. This deviation is of course understood as resulting from the competition between the aging process and the preferential attachment mechanism. According to the preferential attachment mechanism, nodes with a high degree are prone to absorb new links; however, in the present case, getting older reduces the probability of nodes with old links to be selected [12],—since the aging reduces the effective degree of the nodes. This reduces the growth rate of older nodes, whence giving nodes with a smaller degree a higher chance to receive new links.
- The average shortest path to all nodes in a network [67] is known as the closeness centrality measure. It can be seen from Fig 4b that the closeness centrality for the nodes in “our” aged networks is increased to that found for the BA model. Although the centrality of older nodes in aged networks is reduced as compared to the BA model, their centrality is still much larger as compared to younger nodes in the same network.
- The clustering coefficient, Fig 4c, is an indicator that displays the tendency of node’s direct neighbors to connect with each other [19]. Practically, senior members are associated with communities which have a high degree of connectivity, as opposed to recently added members. In other words, although the degrees of old nodes are heavily influenced by the aging process, these old nodes still preserve their strategic position among others. Moreover, it can be observed that old nodes in a network with aging process have a higher clustering coefficient, when compared with those in the same group in the BA model.
- One significant consequence of aging is that new node members have the opportunity to develop into a hub, as opposed to BA model where only early members have a chance of becoming a hub. Hence, the tendency of nodes to connect to their similar nodes, so called assortativity [68] should increase in networks with aged links. This can be seen in the degree-degree correlation palette in Fig 4d–4f. Despite the fact that in the BA model, the largest number of connections are located on hubs and old nodes, in aged networks, a large number of connections can be found between nodes with the same degree.

### Collaboration Network of Oscar winners

As a case study we look at the collaboration network of Hollywood movie actors, which we extract from the Internet Movie Database available at *www*.*imdb*.*com*. We exclude TV movies and TV series from the data, and build the network strictly based on movies for theaters. For each year, we create a network with the condition that two nodes are connected only if the corresponding actors/actresses have at least been cast in a movie together. Thus, we can discuss the network each year. And we can analysis the dynamics of this network through time. Raw data and more information about data extraction technique are available in S1 Text.

**Fig 5:**
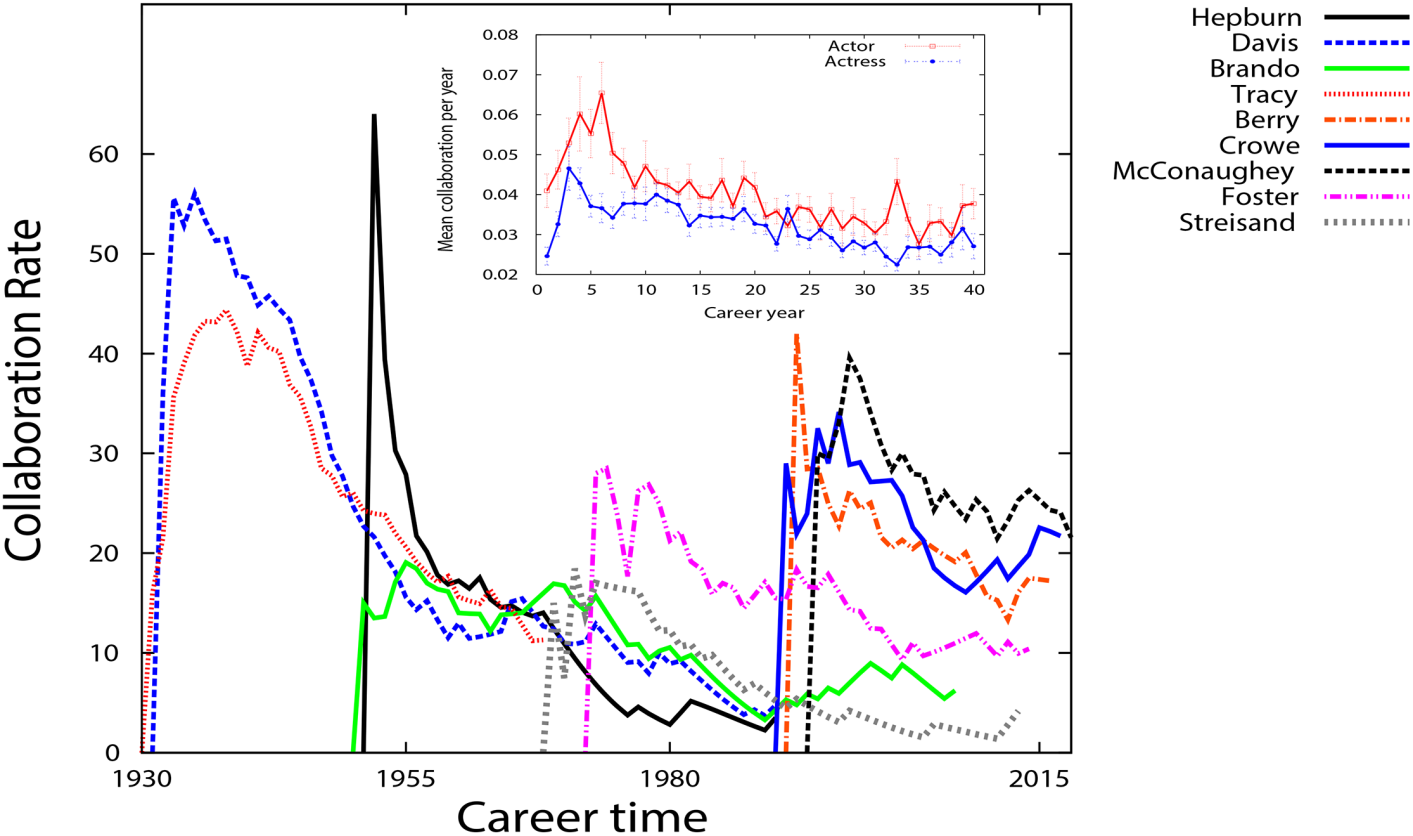
A sample of the total collaboration per year for Oscar winner actors. The “collaboration rate” is the number of staring actors/actresses an actor/actress has played with in a movie each year. It is clear that each actor/actress experiences a peak followed by a long decay in their total collaboration as they age professionally. This peak results from the large number of movies a successful actor/actress has played at the beginning of his/her career before making a name for him/herself. Inlet: the average number of collaborations per year for the 75 Oscar winner actors and 74 Oscar winner actresses. The period, where the actor/actress is making his/her name before the pick, is more visible here. The numbers are normalized by the sum of all collaborations during the career before calculating the average. The rate of collaboration in both curves rapidly increases; after reaching a maximum value, it smoothly decays.

In such a collaboration network, we take a closer look at the collaboration dynamics of Oscar winners actors/actresses. We measure the collaborations of each actor/actress in each year. Fig 5 shows this history for a number of Oscar winners(a gaussian kernel with *σ* = 5 is also applied to data). It can be seen that almost all the actors have the same career pattern. At the start of their acting career, the collaboration rate increases, up to a maximum. After that, the collaboration rate gradually decreases. Despite some exceptions, the general behavior of the collaboration follows the same pattern.

The average collaboration rate of the Oscar winning 75 actors and 74 actresses present a similar pattern to the aging network model, (we averaged history of all actors, starting from their first year career, see inset in Fig 5. Although the general behavior is similar for both curves, it is noticeable that the mean value for the number of collaborations for actresses reaches its maximum sooner than for actors. It is as if the actress dynamics equation has a smaller order of differentiation, *α*, as compared to that for actors collaboration. In other words, it is demonstrated that aging process has a more severe impact on the actresses.

Moreover, if we divide the history of cinema into two equal parts, there is no noticeable difference in dynamical behavior; the mean rate of collaboration in the first and the second half is the same, indicating some reasonable validity (a “stationary system” or “time independence”), thus giving an *a posteriori* argument for the presented model.

Therefore, we can conclude that the above results are in remarkable agreement with findings for the numerical analysis and the subsequently reported simulations in the above sections, in particular in Fig 2.

## Discussion

Correlations between events are intrinsic features of many complex systems. In other words, what happened before an event will impact the current situation: in many cases, the history of a real system cannot be ignored in order to describe its dynamics.

There are models which attempt to include history in a network evolution through a deletion process. Most of these models do so by adding a term to the governing differential equation. Since these additional terms are not unique, there is no clear method to prefer one over the other. Furthermore, such models seem to emphasize that the evolution is much more controlled by the physical environment than by endogenous (social) features. Moreover, often these additional terms do not lead to an analytically tractable solution.

In an attempt to describe these deletion processes on current events and the growth dynamics of networks, we used a kernel function as an averaged measure of the past events within the standard differential equation of the BA model. This approach leads to a fractional order BA differential equation. According to our findings, the presence of fractional order operators imposes a limit on nodes growth, which is in line with the observed behavior of many real system. This suggests that fractional calculus can be a suitable candidate for introducing history and aging in a system.

Whence, by simply generalizing the governing equation in the BA model to an equation with fractional order derivative, a more realistic dynamics for the systems is achieved. As it can be known, in many real systems, such as networks of scientific papers or co-authors, friendship networks, political life, etc., including as discussed movie actors co-staring, the degree of nodes progresses for a certain period of time and then smoothly decays thereafter. This change in topology of the system causes a realistic redistribution of the active importance of the network members.

## Appendix

### Fractional Derivative

For a continuous function *f* on the [*a*, *b*] interval, the left Caputo derivative of order *α* is defined as follow [69]:

$$\begin{array}{c} {}_{a}^{c}D_{t}^{\alpha }[f(t)]=\frac{1}{\Gamma (n-\alpha )}\int_{a}^{t}{\left(t-\xi \right)}^{n-\alpha -1}{\left(\frac{d}{d\xi }\right)}^{n}f\left(\xi \right)d\xi , \end{array}$$ (7)

where *n* is the smallest integer greater than or equal to *α*, *n* = [*α*] + 1. The Caputo derivative has the advantage that in solving fractional differential equations (FDE), it uses integer order boundary or initial conditions.

In attempting to deploy a fractional calculus in the BA model of growing networks, we start with the dynamics equation,

$$\begin{array}{ccc} {}_{t_{0}}^{c}D_{t}^{\alpha }k_{i}\left(t\right) & = & m\frac{k_{i}\left(t\right)}{\sum_{j}^{t}k_{j}\left(t\right)},\\ k_{i}\left(t_{i0}\right) & = & m, \end{array}$$ (8)

where 0 < *α* ≤ 1. For *α* = 1, the above equation becomes the well-known dynamics equation in the BA model. One has a system of fractional order differential equations coupled by the summation in denominator. From here the problem becomes an initial value problem for FDE,

$$\begin{array}{ccc} {}_{t_{0}}^{c}D_{t}^{\alpha }y\left(t\right) & = & f(t,y(t)),\\ y(t_{0}) & = & y_{0}, \end{array}$$ (9)

which can be solved numerically by the predictor-corrector algorithm [70, 71]. Hence, one can reformulate it to an equivalent Volterra integral equation in the form,

$$\begin{array}{c} y\left(t\right)=y_{0}+\frac{1}{\Gamma (\alpha )}\int_{t_{0}}^{t}{\left(t-s\right)}^{\left(\alpha -1\right)}f\left(s,y\left(s\right)\right)ds. \end{array}$$ (10)

To deal with this integral, one can use the product rectangle method, which divides the domain into *n* fragments, *t*_*j*_ = *t*_0_ + *jh*, with equal spaces *h*. The right hand side function, in terms of such a numerical approximation *y*_*j*_ for *y*(*t*_*j*_), is denoted by *f*(*t*_*j*_, *y*_*j*_). Finally, the discrete form reads

$$\begin{array}{c} y_{n}=y_{0}+h^{\alpha }\Sigma_{j=0}^{n-1}b_{n-j-1}f_{j}, \end{array}$$ (11)

with *b*_*n*_ coefficients, which are obtained by using the product rectangle rule in the equispaced case [70, 71],

$$\begin{array}{c} b_{n}=\frac{{\left(n+1\right)}^{\alpha }-{\left(n\right)}^{\alpha }}{\Gamma (\alpha +1)}. \end{array}$$ (12)

These coefficients determine contribution of past dgrees on the current degree. Smaller exponent *α* means smaller *b*_*n*_ coefficients, which leads to faster decay in degree of nodes.

## Supporting Information

S1 Text Collecting data. In order to collect data for Fig 5 we wrote a web crawler to scan Imdb. For each Oscar winner actor/ actress whose name we obtained from Wikipedia, we scanned his/her Imdb page. Each person in Imdb has a page with a list of movies they have participated in with a corresponding release date. For each movie in a person page we scanned the movie page which contains the lead actors in that movie. Since each person has a unique id in Imdb it is relatively simple to check how many people a person has collaborated with in each year. Since movies are multi year projects, we apply a Gaussian kernel to the raw data before plotting individuals in Fig 5.
(ZIP)
